# Allelic Variations of *Plasmodium vivax* Apical Membrane Antigen-1 (*Pv* AMA-1) in Malarious Areas of Southeastern Iran Using PCR-RFLP Technique

**Published:** 2018

**Authors:** Afsaneh MOTEVALLI HAGHI, Sepide MORADI, Mehdi NATEGHPOUR, Gholamhossein EDRISSIAN

**Affiliations:** 1. Dept. of Medical Parasitology and Mycology, School of Public Health, International Campus, Tehran University of Medical Sciences, Tehran, Iran; 2. Center for Research of Endemic Parasites of Iran (CREPI), Tehran University of Medical Sciences, Tehran, Iran

**Keywords:** PvAMA-1, PCR-RFLP, Allelic variations, Iran

## Abstract

**Background:**

Although *Plasmodium vivax* is usually known as benign malaria, some variations of the parasite can result in acute and sever infection. In this study we tried to determine some genetic variations in *Pv*AMA-1 antigen among the samples were collected form southeastern Iran.

**Methods:**

About two ml blood samples were collected into EDTA pre-dosed tubes from 30 *P. vivax*–infected patients individually between 2011 and 2013. A Giemsa stained thick and thin blood film was prepared from each of the patients. A PCR-RFLP technique was employed using EcoR-1, Pvu-II and Hind3 restriction enzymes to determine the allelic variations of the antigen.

**Results:**

A 1300bp gene corresponding to *Pv*AMA-1 was selected for the amplification process. Among the total cases identified in this study 90% showed similar bounds when exposed to the restriction enzymes. Nine isolates (accession numbers: KF435081-KF435083 and JF682785-JF682790) were identified and registered in Gene bank. Identity among isolates was more than 96% in nucleotide level. Dendrogram clarified a close relationship among the clusters in spite of geographical distribution of the parasite.

**Conclusion:**

This study increased our data about prevalence and variation of *Pv*AMA-1 alleles amongst *P. vivax* isolates in southeastern parts of Iran where besides native population bears considerable Afghan and Pakistani immigrants.

## Introduction

Malaria is one of the most common infectious diseases in tropical and sub-tropical regions of the world and still remains as a major parasitic threat to the world population. It is responsible for 438000 deaths in 2015 worldwide (1). Among the malaria parasites, *Plasmodium vivax* is responsible for significant prevalence of *vivax* malaria in the world, with an estimation of 13.8 million cases totally in 2015 (1, 2). Although morbidity and mortality of *P. vivax* are less than *P. falciparum,* the parasite is more widespread than *P. falciparum* in the world particularly outside of the Africa continent. *P. vivax* malaria is considered as a benign parasite but it affects enormous socio-economic impacts among the affected individuals. Moreover, there are some reports of sever vivax malaria cases with considerable clinic- pathological damages (3).

Treatment failures of the infection as a result of drug resistance in some areas encounters control of the disease with a crucial problem (4, 5). Studies on the genetic variations in *P. vivax* can guide us to predict the future situations of different strains of the *Plasmodium* in view of treatment, control and drug resistance. Such studies also help researchers to prepare confident fields for vaccine studies. Considering the different antigens in *P. vivax* is the main pathway to reach the mentioned aims. Among the *P. vivax* antigens apical membrane antigen 1 (*Pv*AMA-1) is worth wile to be considered due to its important role in the parasite to attack host erythrocytes.

The gene of *Pv*AMA-1 protein is expressed during merozoite formation in development of schizont stage that consists of 562 amino acids and 19 cysteines. Unlike other *Plasmodium* antigens, this gene did not show any regional repetitive sequences. Moreover, *Pv*AMA-1 has shown limited genetic diversity in other geographical regions (6). The mentioned antigen can be suggested as a suitable candidate for anti-plasmodia vaccine. This gene includes several alleles that make this region proper marker for typing the parasite populations (7, 8). In some studies, the PvAMA-1 gene was considered in samples collected from malarious areas of Iran. The authors used sequencing method to find haplotypes and diversity in the gene. That results will be compared with our results in the relevant section (9, 10).

In this study, we tried to determine some genetic variation in *Pv*AMA-1 antigen among the samples collected from malarious areas of southeastern Iran.

## Materials and Methods

### Study area

Blood samples were collected from residents of malaria-endemic areas in southeastern Iran nearby the Persian Gulf coast and borderlines of Afghanistan and Pakistan countries. Overall, 30 samples were collected from *P. vivax-*infected patients between 2011 and 2013.

### Blood collection and microscopic examinations

Two ml blood sample was collected into EDTA pre-dosed tube from each *P. vivax* infected patient.

The sample was employed for preparing thick and thin blood smears for microscopic examination and the rest was frozen at 70 °C for PCR analysis. The smears were stained with Giemsa based on WHO malaria microscopy guideline (11). These smears were used for double check to confirm the first detection as well to determine any mixed infection as exclusion criteria for the purpose of the study.

Before to the sampling a written consent according to medical research ethics form, prepared by Medical Research Ethics Committee of Tehran University of Medical Sciences was completed by patient or her/his guardian in children.

**DNA Extraction**: DNA was extracted from the whole blood using genomic DNA extraction kit (BIONEER) according to instructions of the kit. The quality and quantity of extracted DNA were checked using electrophoresis on 1% agarose gel.

A 1300bp gene fragment corresponding to *P. vivax* apical membrane antigen-1 was amplified using the following primers: Forward (5′-ACCGTTGAGAAGCACGA-3′) and Reverse (5′ GATTAGTAGCATCTGCTTGTTCGA-3′). These primers were designed based on the sequence of *P. vivax* sal-1 apical merozoite antigen-1(accession NO: XM-001615397) covered the three regions Domain (I, II, III) of the PvAMA-1 gene. The materials were used for PCR in total volume of 25ul-reaction were as followed: 50 ng extracted DNA as template, 20 pmol of each primer, 12.5ul of PCR master mix (Roche) and ddH_2_o up to 25ul. The target gene was subjected to 30 cycles of amplification. The cycles comprised of denaturation at 95 °C for 20 min, annealing at 58 °C for 30 min and an extension at 72 °C for 2 min. Then, the PCR products were checked by electrophoresis with 1% agarose gel for purification of *Pv*AMA-1 PCR reaction done with 100ul volume. The purified *Pv*AMA-1 gene was sent for sequencing after gel extraction processing using QIA quick Gel Extraction Kit.

### RFLP

In order to determine the presence of different alleles of *Pv*AMA-1 gene in the region, PCR–RFLP technique was done to digest the gene using three restriction enzymes EcoR-1, Pvu-II and Hind3 (Thermo cat No #ER0271, #ER0631 and ferments cat No #ER0501 respectively) according to the manufacturer’s recommendations. The products were visualized under UV illumination after electrophoresis on 3% agarose gel containing ethidium bromide. DNA marker (Roche) was used for comparison of products sizes.

**Sequencing**: Finally 9 samples were sent for sequencing. Nucleotide submitted in GenBank were compared with Sal1, S3 (Indian) and Sk0814 (Korean) isolates. The level of identity and correlation among samples were determined using clustalW2 and MEGA 6 soft wares.

## Results

**Fig. 1:**
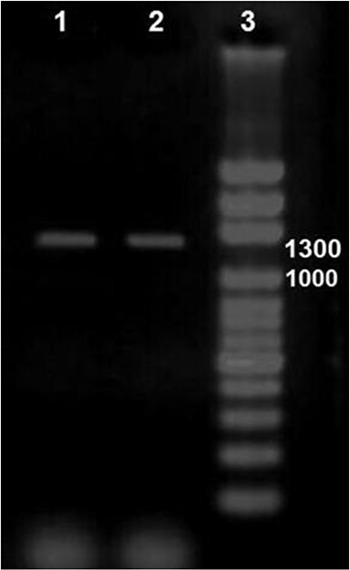
Electrophoretic product of PvAMA-1 gene using PCR technique including columns 1&2 amplified PvAMA-1 gene with 1300bp and DNA size marker with 1000bp in Column 3

Thirty microscopically confirmed *P. vivax* samples were examined for this study. A 1300bp band corresponding to *Pv*AMA-1 gene was detected after amplification process (Fig. 1).

**Fig. 2:**
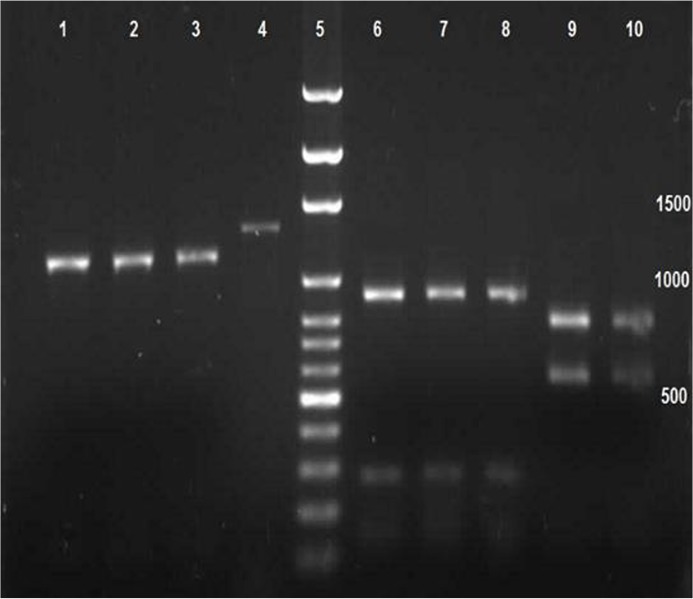
Electrophoretic product of PvAMA-1 gene treated with EcoR I, PvuII, and HindIII, enzymes using RFLP-PCR technique including Columns 1,2&3 treated with EcoRI. Column 4 main bound of *Pv*AMA-1. Column 5 size marker with 1000bp. Columns 6, 7 and 8 treated with PvuII. Columns 9&10 treated with HindIII

Nine isolates were identified and registered in GenBank (accession numbers: KF435081-3 and JF682785-90). The isolates included 402–422 amino acids with 16 residuals cysteine amino acids. All of the 30 PCR products were treated by EcoRI, HindIII and PvuII enzymes. EcoR1 exposed *Pv*AMA-1 created three segments of DNA including 49 bp, 140 bp and 1149 bp bounds. Moreover, exposure of the gene to HindIII and PvuII resulted in 148 bp, 276 bp and 914 bp bounds when digest with the first enzyme and 518 bp and 690 bp bounds via the second enzyme. Among the total cases identified in this study, 90% showed similar bounds when exposed to the above-mentioned enzymes. However, three of them denied resulting in any segmented bound by Hind3 enzyme (Fig. 2).

**Fig. 3:**
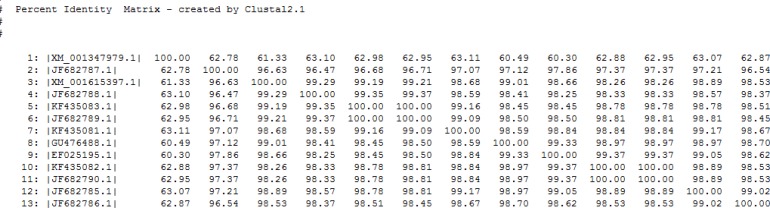
The degree of identity for *P.vivax* AMA-1 gene among isolates from Iran (accession numbers: KF435081-3 and JF682785-90), Sal 1(XM_001615397) from Us, S3 (EF025195.1) from India and SK0814 (GU476488.1) from Korea in comparison with *P. falciparum 3D7* AMA-1 gene (XM_001347979.1) as an outgroup

Moreover, amongst registered isolates in GenBank, Hind3 enzyme could not cut KF435082 isolate. Amplified genomic segment was consisting of three Domains (DI, DII, and DIII) that were compatible with those data recorded in GenBank. Identity among isolates was more than 96% in nucleotide level. Some of them showed up to 99% identity with Sal1 and Indian isolates, while this rate was 60%–63% in comparison with *P. falciparum* as an outgroup (Fig. 3).

**Fig. 4:**
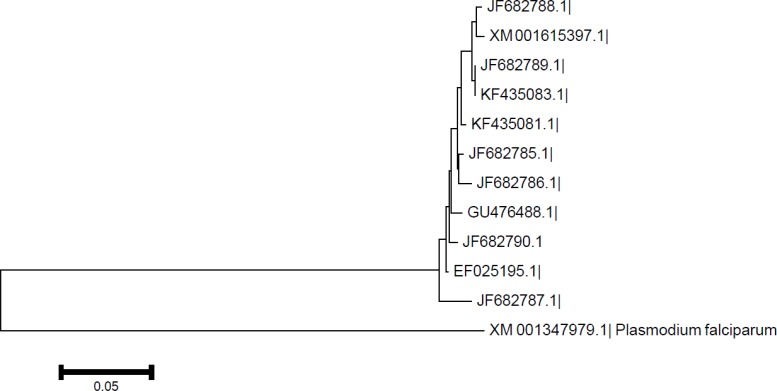
Dendrogram of *P. vivax* isolates taken from the present study and the other isolates from Us (XM_001615397), India (EF025195.1) and Korea (GU476488.1) in comparison with *P. falciparum 3D7* AMA-1 gene (XM_001347979.1) as an outgroup based on sequence alignments of the mentioned samples.

Dendrogram of the samples illustrated that the arrangement of the clusters is close to each other when considered geographical distribution of the parasite (Fig. 4).

## Discussion

Although *P. vivax* is usually known as benign malaria, sometimes can cause acute and sever infection (4). Owing to relapse phenomenon in *P. vivax*, combatting the infection including prevention and treatment takes reasonably long time besides consuming plentiful expenses.

Emerging chloroquine-resistant strains of *P. vivax* in some malarious areas (5) made control of the infection more difficult than before. Although feasibility of producing a successful vaccine against *P. falciparum* and *P. vivax* is debatable at the present time, various studies in the field of vaccine particularly concerning apical membrane antigen (AMA-1) in both species are valuable studies (12). Either similarities or diversities among the different isolates of Plasmodia antigens in various areas can guide us to decide about reasonable methods for control of malaria. *Pv*AMA-1 is expressed in all merozoites of *P. vivax* strains (13).Geographical polymorphism of the gene is limited, but it is important in haplotype activation (14).

In the present study RFLP technique was used due to finding specific areas for DNA fingerprinting, and also for finding restriction cleavage patterns. Moreover, such technique was employed to determine the length of DNA fragment using agarose gel electrophoresis.

In this study, 30 *P. vivax* infected samples collected from malarious areas of southern Iran bordering with Pakistan and Afghanistan countries were considered to determine polymorphism of *Pv*AMA-1.

Out of 3 identified domains for PvAMA-1, domain I included most single nucleotide polymorphism (SNP) than two others, similar to those were reported by a number of authors (15–19), while domain II showed maximum homology either among the isolates or when compared with sal-1, S3 (Indian) and Sk0814 (Korean) isolates.

Domain I and Domain II in *Pv*AMA-1 have been known as immunological protectors against malaria infections in malaria-endemic areas as well as a trustable candidate for antimalarial vaccine (20). Domain III is distinctly responsible in cross-reactivity among the plasmodia species (13).

Stable formation of produced proteins and their specific antibodies, in the studied antigen firmly depends on the relevant cysteine (21) that thoroughly were protected in Iranian studied isolates. Antigenic homology among the studied isolates was 96% or more, indicating high relationship amongst southeastern Iranian *P. vivax* isolates. Moreover, such antigenic homology also occurred amongst the Iranian studied isolates and Sal1, India and Korea isolate. Dendrogram illustrated such correlation among *P. vivax* isolates AMA-1 gene, In fact, some of the isolates from the present study was close to Sal1 and some of them was neighboring with Indian or Korean isolates, while showed lower identity in comparison with AMA-1 gene of *P. falciparum* 3D7 isolate above mentioned. Therefore, these results showed a strong correlation among different isolates for this gene in diverse geographical distributions. These data can help relevant managers to produce some new strategies or control activities to combat against the problem.

The similar homology was found between Iranian and Sal-1 isolates. The rate of homology in Iranian isolates in comparison with Indian and Korean isolates was more than that, such correlation maybe seen due to geographical distribution of the parasite in the region of the world.

A similar study showed 93% homology among 20 isolates collected from northern Brazil (22). Overall, 58 *P. vivax* samples were collected from Iran, and found 23 new haplo-types in parasite population and implied that there is high level of allelic diversity at the domain I of PvAMA-1 among the isolates. In another study on 37 samples, 29 haplotypes were found, but most of the detected mutations were located outside B-cell epitopes, so they reported limited antigenic diversity were present in this antigen (9–10).

*Plasmodium* population in Iran is usually targeted with Afghan and Pakistani Plasmodia isolates due to frequent imported cases by those nationalities but despite this, diversity of *Pv*AMA-1 is not as high as Merozoite Surface Antigen (MSP) in Iran (23). Therefore, such capacity in *Pv*AMA-1 makes the antigen a suitable candidate for vaccine and even for diagnostic marker (24).

## Conclusion

This study increased our data about prevalence and variation of *Pv*AMA-1 alleles amongst *P. vivax* isolates in southeastern parts of Iran where besides native population bears considerable Afghan and Pakistani immigrants. A close correlation was seen in the arrangement of the clusters despite the consideration of geographical distribution of the parasite.
